# INI1/hSNF5-interaction defective HIV-1 IN mutants exhibit impaired particle morphology, reverse transcription and integration *in vivo*

**DOI:** 10.1186/1742-4690-10-66

**Published:** 2013-06-24

**Authors:** Sheeba Mathew, Minh Nguyen, Xuhong Wu, Achintya Pal, Vaibhav B Shah, Vinayaka R Prasad, Christopher Aiken, Ganjam V Kalpana

**Affiliations:** 1 Department of Genetics, Albert Einstein College of Medicine, Bronx, NY, 10461, USA; 2 Department of Microbiology and Immunology, Albert Einstein College of Medicine, Bronx, NY, 10461, USA; 3 Department of Pathology, Microbiology and Immunology, Vanderbilt University School of Medicine, A-5301 Medical Center North, Nashville, TN, 37232-2363, USA

**Keywords:** INI1, IN, HIV-1, Host factors, Morphogenesis, Reverse transcription, Integration

## Abstract

**Background:**

Retroviral integrase catalyzes integration of viral DNA into the host genome. Integrase interactor (INI)1/hSNF5 is a host factor that binds to HIV-1 IN within the context of Gag-Pol and is specifically incorporated into HIV-1 virions during assembly. Previous studies have indicated that INI1/hSNF5 is required for late events *in vivo* and for integration *in vitro*. To determine the effects of disrupting the IN-INI1 interaction on the assembly and infectivity of HIV-1 particles, we isolated mutants of IN that are defective for binding to INI1/hSNF5 and tested their effects on HIV-1 replication.

**Results:**

A reverse yeast two-hybrid system was used to identify INI1-interaction defective IN mutants (IID-IN). Since protein-protein interactions depend on the surface residues, the IID-IN mutants that showed high surface accessibility on IN crystal structures (K71R, K111E, Q137R, D202G, and S147G) were selected for further study. *In vitro* interaction studies demonstrated that IID-IN mutants exhibit variable degrees of interaction with INI1. The mutations were engineered into HIV-1_NL4-3_ and HIV-Luc viruses and tested for their effects on virus replication. HIV-1 harboring IID-IN mutations were defective for replication in both multi- and single-round infection assays. The infectivity defects were correlated to the degree of INI1 interaction of the IID-IN mutants. Highly defective IID-IN mutants were blocked at early and late reverse transcription, whereas partially defective IID-IN mutants proceeded through reverse transcription and nuclear localization, but were partially impaired for integration. Electron microscopic analysis of mutant particles indicated that highly interaction-defective IID-IN mutants produced morphologically aberrant virions, whereas the partially defective mutants produced normal virions. All of the IID-IN mutant particles exhibited normal capsid stability and reverse transcriptase activity *in vitro*.

**Conclusions:**

Our results demonstrate that a severe defect in IN-INI1 interaction is associated with production of defective particles and a subsequent defect in post-entry events. A partial defect in IN-INI1 interaction leads to production of normal virions that are partially impaired for early events including integration. Our studies suggest that proper interaction of INI1 with IN within Gag-Pol is necessary for proper HIV-1 morphogenesis and integration.

## Background

Retroviruses express Gag, Gag-Pol and Env as polyproteins. During late events, retroviral Gag and Gag-Pol polyproteins assemble together within the producer cells. Cleavage of the precursor polyproteins by the viral protease during maturation leads to infectious virions [1-3]. While Gag is the structural component essential for assembly, genetic studies over more than a decade have demonstrated that mutations in the Pol component of the Gag-Pol polyprotein leads to defects in morphogenesis of particles that can manifest either as defects in assembly and particle production or as defects in subsequent rounds of infection. In HIV-1 and other lentiviruses, Pol protein consists of protease (PR), reverse transcriptase (RT) and integrase (IN). IN catalyzes the integration of viral DNA into host chromosomal DNA by sequential steps of 3’ processing and strand transfer [4]. Deletions or certain point mutations of IN (class II IN mutations) while not affecting the catalytic activity of integrase, lead to defects in stages other than integration (termed pleiotropic effects) including viral protein synthesis/stability, assembly and virus particle release, uncoating, reverse transcription and nuclear localization [5-15]. Similarly, certain mutations in RT have been shown to significantly reduce assembly and virion release [16-18]. These results suggest that perturbing the Pol portion of Gag-Pol can lead to defects in assembly, morphogenesis and particle release, which can in turn lead to production of non-infectious particles. However, the role of Pol in mediating these late event defects or the mechanism of these pleiotropic effects is poorly understood. One possibility is that mutations in Pol could interfere with Gag/GagPol multimerization or proteolytic processing, giving rise to multiple effects [16,19]. Another possibility that has not been explored is that IN and RT mutations may alter the interaction of Pol with cellular proteins leading to defects in various stages of HIV-1 replication.

Several cellular proteins interact with IN and RT. We isolated the first IN-interacting cellular protein termed Integrase interactor (INI)1/hSNF5 [20]. INI1 selectively binds to HIV-1 IN within the context of Gag-Pol and is specifically incorporated into HIV-1 virions during assembly in the producer cells [21,22]. INI1/hSNF5 has three highly conserved domains, the first two are imperfect repeats of each other (termed Rpt1 and Rpt2) and a third domain termed homology region III (HR III) [23]. The Rpt1 domain is the minimal IN-interaction domain of INI1 and it is necessary and sufficient to bind HIV-1 IN [23]. The Rpt2 domain harbors a masked nuclear export signal (NES), which when unmasked, allows nuclear export of INI1 [24].

We have shown that INI1 plays a role in the late events of HIV-1 replication including assembly and particle production. Expression of a minimal IN-binding fragment of INI1, termed S6, inhibits HIV-1 particle production in a dominant negative manner [21]. The expression of S6 in HIV-1-producing cells leads to the disruption of the IN-INI1 interaction resulting in a potent (1000 to 10,000 fold) inhibition of HIV-1 particle production [21]. Single amino acid substitutions in either S6 or IN that abolish the mutual protein-protein interaction, abrogate the inhibition [21]. Furthermore, deletion of IN from Gag-Pol and expressing IN as a Vpr-IN fusion *in trans*, abrogates S6-mediated inhibition. These results indicate that the S6 directly binds to IN within Gag-Pol and interferes with late stages of HIV-1 replication [21]. In a separate study, we showed that S6 fragment inhibits the post-translational early stages of HIV-1 assembly such as Gag/Gag-Pol trafficking and processing [25]. Furthermore, human cell lines lacking INI1 exhibit defects in HIV-1 particle production, and the expression of INI1 in these cells restores particle production [26]. Together, these studies highlight the importance of Gag-Pol interaction with INI1 for proper assembly and particle production.

INI1/hSNF5 is a core component of the SWI/SNF ATP-dependent chromatin-remodeling complex and modulates integration activity *in vitro*[20]. Because of its association with SWI/SNF, INI1 is thought to target integration into open chromatin regions [20,27]. Moreover, recent studies demonstrate that INI1 within the SWI/SNF complex is required for integration of HIV-1 DNA into chromatinized DNA targets *in vitro*[28].

INI1/hSNF5 is incorporated selectively into HIV-1, but not into other retroviral particles, and IN is required for this incorporation [22]. Binding of IN to INI1 also leads to recruitment of other INI1 binding proteins into HIV-1 virions. INI1 binds to SAP18 (Sin3a associated protein of 18 KD), a component of the Sin3a-HDAC1 complex. In addition to INI1, components of the Sin3a/HDAC1 repressor complex, including SAP18 and HDAC1, are incorporated into HIV-1 virions [29]. HIV-1 particles containing the catalytically inactive HDAC1^H141A^ mutant show reduced deacetylase activity and reduced infectivity. The block due to the presence of the HDAC1^H141A^ mutant appears to be at or prior to reverse transcription in the target cells [29]. These observations are corroborated by the findings that producer cells lacking INI1 show greatly reduced HIV-1 particle production and that the few particles produced are defective for reverse transcription [26]. These studies indicate that interactions of INI1/hSNF5 and associated complex such as HDAC1 in producer cells are necessary for HIV-1 particle production and for the infectivity of those particles. These studies collectively suggest that a defect in INI1 binding could cause various defects during HIV-1 replication including assembly and particle production, reverse transcription and/or integration.

To further investigate the role of the IN-INI1 interaction in HIV-1 replication, we isolated and characterized mutants of IN impaired for binding to INI1 using a reverse yeast two-hybrid system. We characterized a panel of mutants obtained in this screen by studying their effect on various steps of HIV-1 replication. Our analyses indicate that many mutants highly defective for INI1 binding are severely compromised for replication *in vivo* and are defective for early post-entry reverse transcription. These mutants exhibit defective virion morphology. Other mutants, partially defective for INI1 binding, although competent for reverse transcription, are partially impaired for integration. Together with previous findings, our results suggest that the association of INI1 with IN within the context of Gag-Pol is necessary for the proper morphogenesis and for the production of virion particles competent for infection.

## Results

### Isolation and characterization of INI1-interaction-defective (IID) IN mutants

**Figure 1:**
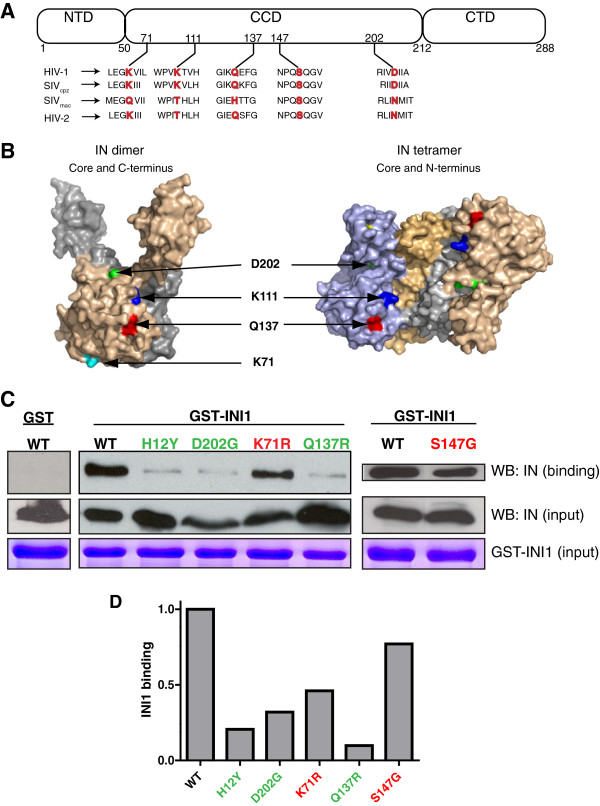
**IID-IN mutants map to surface of IN crystal structures and exhibit defects in binding to INI1 *in vitro: *A.** A cartoon illustrating the positions of IID-IN mutations on three domains of IN (not drawn to scale). NTD = N-terminal domain; CCD = Central core domain; CTD = C-terminal domain. Sequence spanning the mutant residues from HIV-1 and three other related lentiviruses are indicated below the cartoon, and the parent residues that are mutated is shown in red. **B.** Mutant residues with high surface accessibility are highlighted on Pymol rendition of two domain crystal structures of IN. The left diagram illustrates the structure of a dimeric core + C-terminal domains (PDB: 1EX4); and the right diagram illustrates the tetramer formed by the core + N-terminal domains (PDB: 1K6Y). Residues are indicated in the following colors: D202- green, K111- blue, Q137- red, K71- cyan. Monomers within the IN dimer and tetramer are represented by different colors. **C.** IID-IN mutants are defective for binding to INI1 in vitro. *In vitro* GST pull-down assays were performed by using 293 T cell lysates expressing YFP-WT IN or YFP-IID-IN mutants. Lysates were normalized for YFP-IN (WT or IID-IN mutant) expression and bound to GST-INI1 immobilized onto glutathione sepharose beads. GST-INI1 input was measured by Coomassie stain. WB = Western blot. **D.** Graphic representation of degree of binding of each YFP-IID-IN mutant relative to that of YFP-WT, as measured by densitometric analysis. Amount of YFP-IN bound was normalized against amount of YFP-IN loaded.

To determine the consequences of disrupting the IN-INI1 interaction on HIV-1 replication, we sought to isolate mutants of IN that are defective for binding to INI1. Mutants were selected using a reverse yeast reverse two-hybrid system. A random mutation library of IN fused to GAL4 activation domain (GAL4AC-IN*, [30]) was screened against the wild type full-length INI1 fused to LexA DNA binding domain (LexADB-INI1). As expected, INI1 wild type controls interacted strongly to wild type IN, resulting in *LacZ* expression and blue colonies. Screening of mutant IN library against LexADB-INI1 resulted in several white colonies. Plasmids expressing GAL4AC-IN* mutants were isolated from these colonies and were retransformed into yeast along with LexADB-INI1 to confirm their interaction-defective phenotype. These GAL4AC-IN* clones were also tested for their ability to dimerize with LexADB-IN. Those clones that retained the interaction with LexADB-IN (thus able to dimerize) but lacked the interaction with LexDB-INI1 were selected for further analysis and were termed **I**NI1-**I**nteraction **D**efective-IN clones (IID-IN). Sequence analysis of eleven clones isolated from this screen, which were defective for binding to INI1 but not for binding to IN, revealed the presence of one or more substitution mutations (Additional file 1: Table S1). The mutated residues spanned all domains of IN, with most mapping to the core domain. Among these mutations the previously published INI1-interaction defective mutant, K71R, that retains 40% binding to INI1, was also identified (see Table 1, [31]). Since surface residues are most likely to be engaged in protein-protein interactions, we analyzed the surface accessibility of the parent residues that were mutated in the IID-IN clones by using the Protein Interfaces Surfaces and Assemblies (PISA) program [32]. Five different mutated residues that showed high surface accessibility (>50) were chosen for further analysis (Table 1)*.* A previously characterized IN mutant, H12Y, was used as a control in all the subsequent experiments (the H12 residue is not surface accessible). Mapping of surface residues on solved crystal structures of the IN dimer and tetramer (PDB: 1ex4 and 1k6y respectively [33,34]) revealed the presence of parent residues along one face of the IN monomer, implicating this surface as being involved in INI1 binding (Figure 1B). One of the positions, S147, is located on the disordered flexible loop and hence could not be mapped on the crystal structure.

**Table 1:** Surface accessibility (SA) and phylogenetic conservation of IN residues implicated in INI1 binding

	**Surface accessibility**^**a**^						**% identity among lentiviruses**^**b**^	
**HIV-1 IN residue**	**IN dimer**		**IN tetramer**				**HIV-1 and SIV**_**cpz**_	**Non-HIV-1/SIV**_**cpz **_**primate lentiviruses**
	**A**	**B**	**A**	**B**	**C**	**D**		
**K71**	118	79	90	94	99	99	98	85
**K111**	65	81	122	115	138	113	93	32
**Q137**	71	102	59	56	47	56	91	24
**S147**	71	N/A	N/A	N/A	N/A	N/A	100	100
**D202**	71	61	68	50	55	50	100	14

^a^Determined using PISA program based on IN crystal structures of dimer and tetramer (PDB#s 1ex4 and 1k6y, respectively).

^b^Determined using HIV Sequence Compendium (2010).

Since INI1 selectively binds to HIV-1 IN but not to other primate lentiviral or retroviral INs [22], we examined the relative conservation of IID-IN residues among HIV-1 and the closely related SIV_cpz_ strains, or among other primate lentiviruses using the 2010 HIV-1 sequence compendium. All IID-IN parent residues displayed at least 90% identity among HIV-1 and SIV_cpz_ strains, but exhibited varying degrees of conservation among IN proteins from other primate lentiviruses (Table 1 and Figure 1A). Most notably, the residue D202 was invariant among HIV-1 and SIV_cpz_ IN sequences, however, in the majority of other primate lentiviruses, D202 is replaced by asparagine (N). This analysis indicated that residues implicated in INI1 binding are highly conserved among HIV-1 INs but are not as conserved among other lentiviruses.

### IID-IN mutants are defective for INI1 binding *in vitro*

To confirm the interaction defects of IID-IN mutants, a GST-pull down assay was performed using mammalian extract containing YFP-WT-IN or YFP-IID-IN normalized for YFP-IN levels, and bacterially expressed GST-INI1 bound to G-beads. The results of the pull down assay confirmed that all IID-IN mutants were defective for binding to INI1. Furthermore, these mutants could be categorized into two groups: (1) Mutants that were highly defective for INI1 binding, including H12Y (previously identified), Q137R, and D202G; and (2) mutants that displayed partial binding, including S147G and K71R (Figure 1C and D). K111E failed to express high enough protein levels in mammalian cells to be tested for INI1 binding in this system. However, *in vitro* binding of His_6_-K111E with GST-INI1 revealed ~60% binding compared to His_6_-WT (data not shown).

### HIV-1 viruses harboring IID-IN mutants are defective for replication

**Figure 2:**
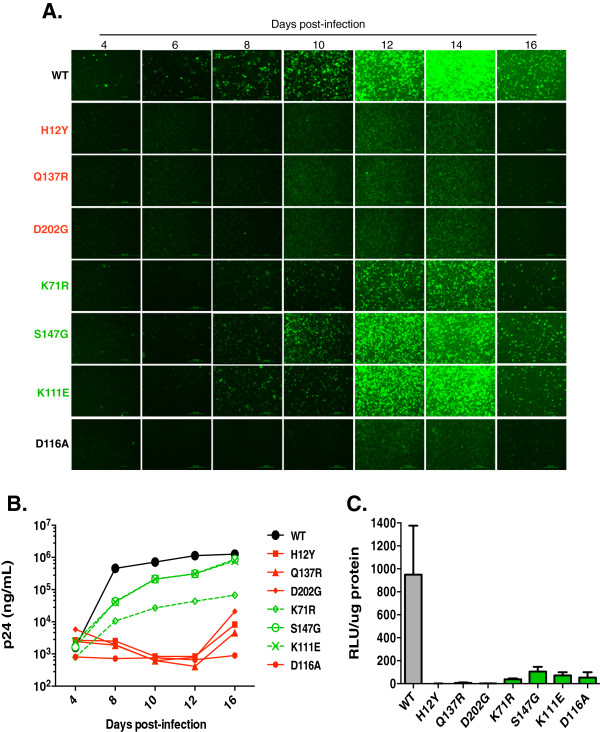
**IID-IN mutants are defective for multi-round and single-round replication: A.** Fluorescence microscopic images of CEM-GFP cell cultures infected with HIV-1_NL4-3_ carrying WT IN or IID-IN mutants in a multiday infection. CEM-GFP cells were infected with normalized amounts of HIV-1_NL4-3_ (25 ng p24) WT or IID-IN mutants. Cultures were imaged for expression of LTR-GFP every 48 hr over a 16-day course of infection. **B.** Measurement of virus particle release in the culture supernatant by p24 ELISA. Culture supernatants were collected from CEM-GFP cells infected with normalized amounts of HIV-1_NL4-3_ virus harboring WT or IID-IN mutant IN, every 48 hr and measured for p24 levels. Green = IID-IN mutants partially defective for INI1 binding; Red = IID-IN mutants highly defective for INI1 binding. Data is representative of two independent experiments. **C.** Infectivity of HIV-Luc virus harboring WT IN or IID-IN mutations. Graph illustrates luciferase activity induced by infection of HeLa cells with HIV-Luc WT and IID-IN mutants in a single round of infection. HeLa cells were infected with 5 ng p24 of WT or IID-IN mutant HIV-Luc. The infectivity was quantified by measuring luciferase activity 24 hours post-infection. Data represents average of three independent experiments (*Mean +/- SEM*).

To determine the effect of IID-IN mutants on HIV-1 replication, these mutants were incorporated into the full-length HIV-1_NL4-3_ molecular clone, and the wild type and mutant virions were tested for their ability to replicate in multi-day replication assays. Mutant virions were produced in 293 T cells by transfection. Measurement of total intracellular and virion-associated capsid levels revealed no significant defect in level of p24 production (Additional file 2: Figure S1). Normalized amounts of NL4-3 WT and mutant virions (25 ng of p24) were used to infect CEM-GFP cells, a reporter T-cell line containing integrated LTR-GFP. Viral replication was monitored over a course of 16 days by observing cellular GFP expression levels and quantifying p24 in the culture supernatant. GFP expression of cultures infected with WT NL4-3 reached a maximum level at 14 days, indicating that viral replication peaked at around this time (Figure 2A). Viruses harboring H12Y, Q137R, and D202G, IID-IN mutations that were severely impaired for INI1 binding were highly defective for replication as very little GFP expression above basal levels was observed at peak points in these cultures (Figure 2A). While the replication of mutants K71R, S147G, and K111E, which bore partial binding to INI1, peaked at 14 days, the overall GFP levels were lower than that of cells infected with WT virus, indicating a partial defect in replication of these viruses.

Measurement of p24 levels in the infected CEM-GFP culture supernatants corroborated the GFP expression data (Figure 2B). H12Y, Q137R, and D202G, mutants that were highly defective for inducing GFP expression, exhibited background levels of p24 in the culture supernatants. K71R, S147G and K111E mutants that exhibited a partial defect in inducing GFP expression, demonstrated a partial defect in p24 production. These mutants expressed very low levels of p24 that steadily rose during the course of infection, but overall lower than that of the WT. After about 12 days, the partially defective mutants displayed an increase in p24 production. Our analysis of the peak cultures (after 16 days) from one of the partially defective mutants, S147G, indicated that the IN mutation in these virions had been reverted to wild type (data not shown). Of the partially defective mutants, K71R appeared to be the most defective in multi-round replication, however, the p24 levels of this mutant also rose during the infection time course and reached levels above background. Taken together, these studies indicated that all IID-IN mutants exhibited defects in multi-day replication.

To determine if the reduced infectivity of the mutant viruses is due to a defect in early events, a single cycle infection assay using a VSV_G_ pseudotyped HIV-Luc reporter virus was performed. In this system, infection and integration of HIV-Luc virus leads to expression of the luciferase gene, allowing for measurement of infectivity. HeLa cells were infected with normalized amounts of WT and IID-IN mutants (5 ng p24) for 24 hours, cells were harvested after infection and total protein and luciferase activity was determined. Corroborating the multi-round replication data, all mutants highly defective for binding INI1 were severely (200-1,000 fold compared to WT) impaired for infection, while mutants bearing partial INI1 binding were partially (~2-20 fold compared to WT) defective for infection (Figure 2C). Taken together, these data suggested that the degree of defect in INI1 binding was reflected in the degree of replication impairment.

### Highly-defective IID-IN mutants are impaired for early and late reverse transcription *in vivo*

The data from the single-round replication assay suggested that the block to infection of IID-IN mutants occurred at some step during early events. Since mutants of both HIV-1_NL4-3_ and HIV-Luc, which carry different envelope proteins and have different modes of entry, were defective for infection, we surmised that the block in replication was unlikely to occur at entry. We therefore tested for the ability of these mutants to undergo post entry events including early and late reverse transcription (RT), nuclear localization, and integration to identify the stage at which these mutants were blocked during replication *in vivo*.

**Figure 3:**
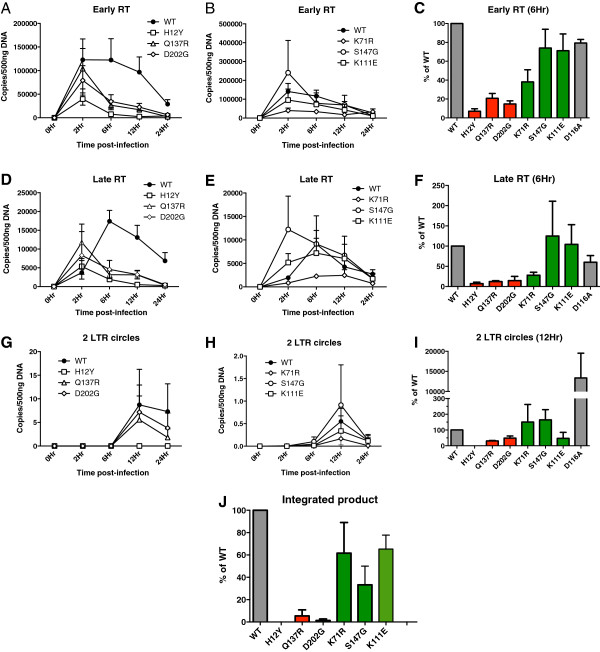
**IID-IN mutants are defective for early post entry events. A-C.** Effect of IID-IN mutations on early RT. Using genomic DNA from 293T cells infected with HIV-Luc harboring WT-IN, IID-IN, or catalytic mutant D116A, early RT products were quantified by qRT-PCR at indicated times post-infection. Effects of highly defective IID-IN mutants on early RT are shown in **(A)** and partially defective mutants in **(B)**. **C.** Relative effect of IID-IN mutations on early RT as compared to WT. Data represents amounts at 6 h post-infection averaged from three independent experiments (Mean +/- SEM). **D-F.** Effect of IID-IN mutations on late RT. As in panels A-C, late RT products in infected cells were quantified via qRT-PCR at indicated times post-infection. Effects of highly defective IID-IN mutations on late RT are shown in **(D)** and partially defective mutants in **(E)**. **F.** Relative effect of IID-IN mutants on late RT as compared to WT. Data represents amounts at 6 h time post-infection averaged from three independent experiments (Mean +/- SEM). **G-I.** Effect of IID-IN mutations on formation of 2LTR circles. 2LTR products in infected cells were quantified via qRT-PCR at 12 h post-infection. The data represents average of three independent experiments (Mean +/- SEM). Effects of highly defective IID-IN mutations on 2LTR circle formation are shown in **(G)** and partially defective mutations in **(H)**. **I.** Relative effect of IID-IN mutations on 2LTR circle formation as compared to WT. Data represents amount of 2LTR products at 12 h post-infection averaged from three independent experiments (Mean +/- SEM). **J.** The effect of IID-IN mutations on integration. Alu-Gag products were quantified in infected cells via qRT-PCR at 24 hours post-infection. Data are compared to WT and represents average of three independent experiments (Mean *+/-* SEM).

To determine the effect of mutations on minus strand strong-stop (early RT) and second-strand transfer (late RT), cells were infected with 20 ng p24 of WT and IID-IN HIV-Luc mutant viruses. Total DNA was collected over a time course of infection (0-24 hours) and subjected to quantitative real time PCR (qPCR) using primers that specifically amplify early or late RT products. The WT virus revealed a sharp increase of early and late RT products at 2 and 6 hours post-infection, respectively (early RT products, Figure 3A and late RT products, Figure 3D). In contrast, H12Y, Q137R, and D202G mutants all showed severe defects in both early and late RT product formation compared to WT (Figure 3A and D). While WT infection led to a clear time-dependent rise and fall of RT products, the highly defective IID-IN mutant RT product levels showed a premature increase at 2 hr. time point and quickly returned to baseline by 6 hr. and subsequent time points during infection (Figure 3A and 3D).

The partially defective IID-IN mutants, K71R, S147G, and K111E were also tested for early or late RT product formation. The partially defective mutants S147G and K111E were not defective for early (Figure 3B) or late RT (Figure 3E). However, K71R mutant exhibited a partial defect in early RT and more significant defect in late RT (Figure 3B and 3E). Analyses of early and late RT products at 6 hrs. post-infection indicated that the highly defective mutants are ~10 fold defective when compared to wild type (Figure 3C and 3F). Among the partially defective mutants, the K71R mutant exhibited an intermediate phenotype, in that it was ~2.5 fold defective for early RT (Figure 3C), and ~3.5 fold defective for late RT (Figure 3F). The remaining two mutants, S147G and K111E, did not exhibit significant defects in early or late RT (Figure 3C, 3F).

### Effect of IID-IN mutations on nuclear localization

We next examined the ability of mutants for nuclear localization, by measuring 2LTR circle formation (Figure 3G-I). 2LTR products peaked at 12 hours post-infection for WT, Q137R and D202G viruses (Figure 3G). The defect was most dramatic for H12Y, which had no detectable levels of 2LTR circles (Figure 3G and I). Analysis of the 2LTR formation by all the mutants at the peak level (12 hrs.) as compared to WT indicated that Q137R, D202G and K111E exhibited partial reduction in 2LTR circle formation (Figure 3I). However, K71R and S147G mutants exhibited no significant defect in 2LTR formation (Figure 3I).

For all the above experiments of determining early and late RT and 2LTR circle formation, we used the Class I catalytic mutant, D116A, specifically defective for integration, as control. This mutant displayed no significant defect in early and late RT, however, exhibited several logs increase in 2LTR circle formation, consistent with previous reports (Figure 3C, F and I). These studies together indicated that while highly defective IID-IN mutants are defective for early and late RT, and consequently for 2LTR formation, the partially defective mutants were not defective for early post entry events.

### Partially defective IID-IN mutants are defective for integration

We next examined the ability of all the mutants for integration *in vivo* using Alu-Gag real-time quantitative PCR (Figure 3J). The two mutants Q137R and D202G were severely impaired for integration; the remaining mutants K71R, S147G and K111E exhibited partial defect in integration that was correlated to their impairment in binding to INI1. We were able to purify S147G and K111E and found that they retained their ability to carry out in vitro strand transfer activities, indicating that the catalytic activity of these mutants was unaffected (data not shown).

### IID-IN mutants display aberrant virion morphology

The above data indicated that while highly defective IID-IN mutants were impaired for early and late RT as well for the subsequent events of replication in the target cells, the partially defective IID-IN mutants were able to pass through early and late RT, but were partially defective for integration. Since INI1 has a role in late events of HIV-1 replication, we surmised that one reason for the observed defects in reverse transcription of IID-IN mutants was due to lack of proper Gag-Pol-INI1 interactions leading to aberrant assembly and maturation of particles, which in turn may lead to defects in post-entry events. To investigate this possibility, we examined the IID-IN mutants for: (i) viral protein processing in the producer cells; (ii) incorporation of RT and IN into virions; and (iii) virion particle morphology using transmission electron microscopy (TEM).

**Figure 4:**
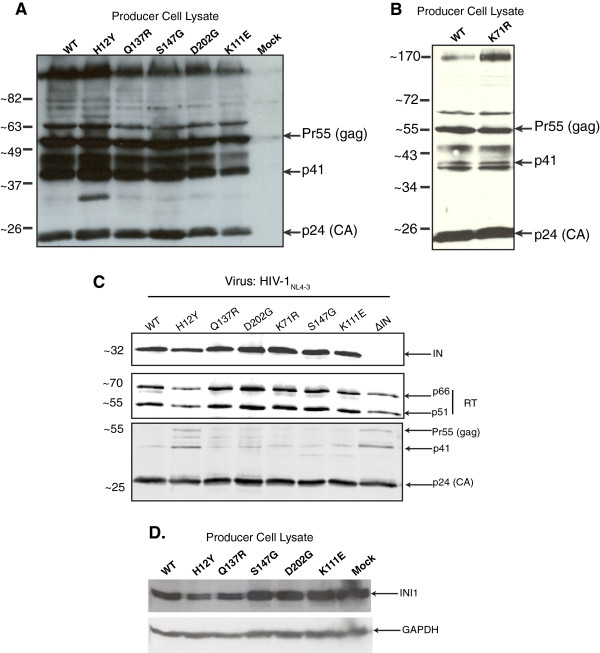
**IID-IN mutants exhibit normal viral protein processing encapsidation of RT and IN into virions: A and B.** Immunoblot analysis of intracellular viral proteins. 50 ug of total protein from 293 T producer cells transfected with HIV-1_NL4-3_ WT or IID-IN mutants were separated on a 15% SDS-PAGE gel and transferred to nitrocellulose. The blots were probed with human patient sera. **C.** Immunoblot analysis of incorporation of viral proteins. Total protein lysates from 350 ng (p24) of HIV-1_NL4-3_ WT or mutant virions were separated by SDS-PAGE, transferred onto nitrocellulose and probed for the presence of IN, RT, and p24 using antibodies specific to each protein. **D.** Immunoblot analysis to determine the levels of endogenous INI1 in cells expressing IID-IN mutant viruses. Total protein from 293 T producer cells transfected with HIV-1_NL4-3_ WT or IID-IN mutants were probed with α-INI1 antibodies. The same blot was probed with α-GAPDH antibody as loading control.

To determine the viral protein processing, total cellular lysates of producer cells transfected with either the wild type or mutant HIV-1_NL4-3_ were probed with α-HIV-1 serum. All IID-IN mutants exhibited WT patterns of viral protein levels and processing in producer cells, except for H12Y (Figure 4A and B), which exhibited the presence of an additional α-p24 antibody-reactive band that migrated between 26 and 37 kDa (Figure 4A). These results indicated that while H12Y mutant exhibits a slightly defective processing, the remaining IID-IN mutants undergo normal protein processing in producer cells.

Immunoblot analysis of mutant virus preparations normalized for p24 revealed incorporation of normal levels of viral proteins into virions, comparable to that of WT (Figure 4C). Except for H12Y, there was no significant decrease in protein levels among IID-IN mutants compared to that of WT, indicating that the dramatic defect in early events is not due to defects in incorporation of IN and RT. The H12Y mutant showed decreased levels of incorporation of IN and displayed higher levels of unprocessed Gag proteins within the virions including Pr55 and Pr41 (Figure 4C). This pattern was similar to the pattern observed in virus deleted for IN (see Figure 4C). Furthermore, there were no appreciable defects in the level of RT proteins. Thus, the defects in reverse transcription among highly defective IID-IN mutants and the integration defect observed in partially defective mutants do not appear to be due to abnormal processing of viral proteins in the producer cells or to low levels of viral proteins within the virions. We also determined the endogenous levels of INI1 in cells expressing IID-IN mutants. As shown in Figure 4D, the endogenous levels of INI1 did not drastically change as a result of expression of HIV-1 harboring IID-IN mutants. Therefore, our results indicate that the defective activity of IID-IN mutants in the cells and viruses reflect their defective interaction with INI1, rather than changes in the levels of IN mutants or INI1 in the host cell.

**Figure 5:**
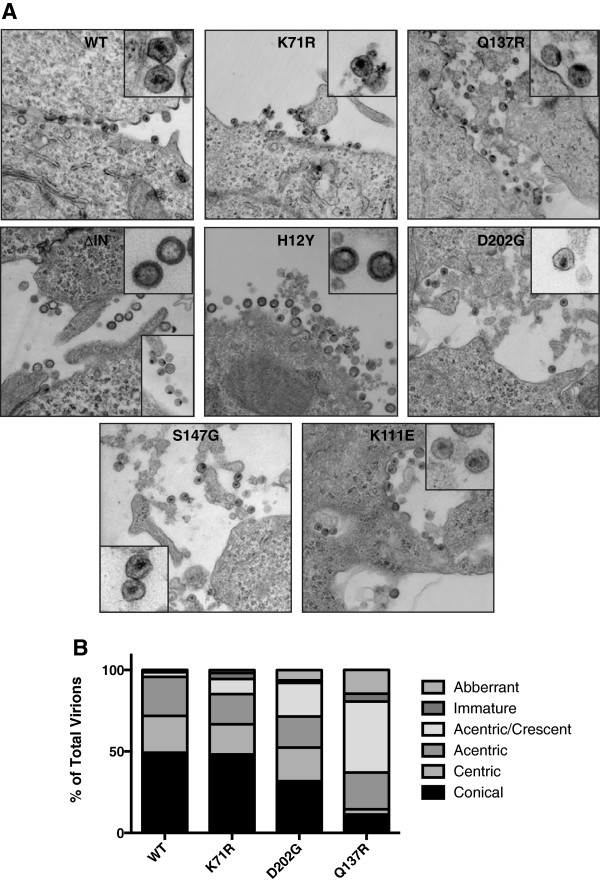
**Ultrastructural analysis of producer cells expressing IID-IN mutants: A.** 293 T cells transfected with pNL4-3 harboring WT or IID-IN mutants were fixed 6 hours post-media change in buffer containing 2.5% glutaraldehyde and post-fixed with 1% osmium tetroxide. Cut sections were imaged on a JEOL 1200EX and images were captured at ×20,000 magnification. Insets are zoom-in images of representative virions. **B.** Quantitation of virion morphologies from various IID-IN mutants. Morphology of 50-70 virions was analyzed for each category of virus, mutant or wild-type.

To determine if the defects observed for the IID-IN mutants correlated with defects in morphology, we carried out ultra-structural analysis of cells producing these virions by using transmission electron microscopy (TEM). We examined 70-100 virions from about 7-12 infected cells each, for the presence of mature and immature virions of different morphology and determined the percentage of virions with conical, centric, acentric, acentric crescent, immature and aberrant capsids. Virions at various stages of budding and morphogenesis were readily observed in cells producing the wild type virions. The mature virions exhibited typical cone shaped or centric core capsid structures (Figure 5A and B). The partially defective IID mutants (K111E and S147G) exhibited normal capsid morphology very similar to that of the wild type (Figure 5A). Interestingly, the highly defective IID-IN mutants exhibited gross defects in morphology. Q137R mutant virions harbored crescent and acentric capsids that were stuck to the viral envelope (Figure 5A and B). The mutant D202G, although demonstrated presence of mature particles, exhibited the presence of significant acentric crescent like particles (Figure 5B). In case of D202G, about 25% of the particles exhibited acentric crescent shaped, or immature or aberrant cores (Figure 5B). H12Y virions exhibited the presence of immature particles, which were very similar to that of ΔIN virions (Figure 5A). These results indicated that the degree of INI1 interaction defects correlated to the severity of the defect in virion morphology.

### The virions harboring IID-IN mutations are not defective for RT activity and uncoating *in vitro*

**Figure 6:**
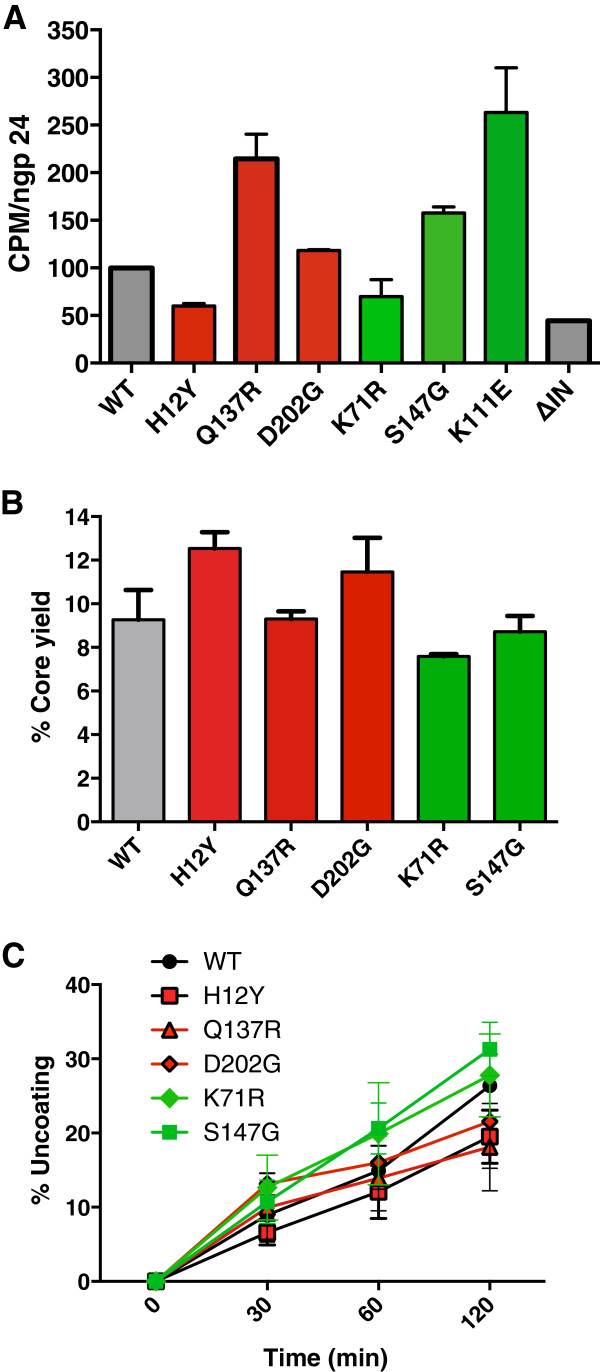
**IID-IN mutant virions are not defective for RT activity and *in vitro *uncoating: A.** Graphic representation of exogenous RT activity in IID-IN mutant virions. Viral lysates from 500 ng p24 each of HIV-1_NL4-3_ WT and IID-IN mutant virions were tested for *in vitro* RT activity using a poly-rA template, oligo-dT primer, and dNTPs in the presence of ^32^P-dTTP. RT activity was determined by incorporation of radiolabeled dTTP and measured by CPM in each reaction. Data represents average of three independent experiments (*Mean +/- SEM*). **B.** Quantitation of CA levels in cores purified from wild type and IID-IN mutant virions. Cores were isolated from concentrated virion suspensions by detergent treatment and density gradient ultracentrifugation, and the levels of core-associated CA were quantified as a percentage of the total CA protein in each gradient. Shown are mean values from three independent experiments; error bars represent SEM. **C.** Kinetic assay of HIV-1 uncoating *in vitro*. Purified cores were incubated at 37°C at indicated times prior to pelleting by ultracentrifugation, and the levels of CA in the supernatants and pellets were quantified by p24 ELISA. Shown are mean values from three independent experiments; error bars represent SEM.

Since IN binding to RT has shown to play a role in reverse transcription and uncoating, a defect in early reverse transcription *in vivo* could be due to defects in reverse transcriptase activity, uncoating or both [14,35,36]. Therefore, to determine these possibilities, the reverse transcriptase activities of IID-IN mutant viruses were tested in an exogenous RT assay using normalized amounts of WT and mutant HIV-1_NL4-3_ virions [22]. We found that all the mutants exhibited significant levels of RT activities *in vitro* indicating that the RT enzyme within the mutant virions is functional (Figure 6A).

We next tested ability of mutants to undergo uncoating by examining alterations in capsid stability *in vitro*. Uncoating assays were carried out *in vitro* with cores isolated from the WT and mutant HIV-1_NL4-3_ particles. HIV-1 cores were purified by the detergent “spin-thru” approach as previously described [37]. Cores were recovered from the gradients and tested in a standard *in vitro* uncoating assay. We found that presence of IID-IN mutations had only small effects on the levels of CA associated with the purified HIV-1 cores (Figure 6B). We next examined the effects of the mutations on uncoating using purified cores. Upon incubation at 37°C, the wild type and mutant cores released CA protein into a soluble form at comparable rates, indicating that the mutants do not exhibit any defects in uncoating *in vitro* (Figure 6C). Collectively, these results indicated that cores isolated from mutant virions were not defective for reverse transcriptase activity and that the mutations did not markedly alter the intrinsic stability of the viral cores.

### IID-IN mutant interactions with other IN binding partners

**Figure 7:**
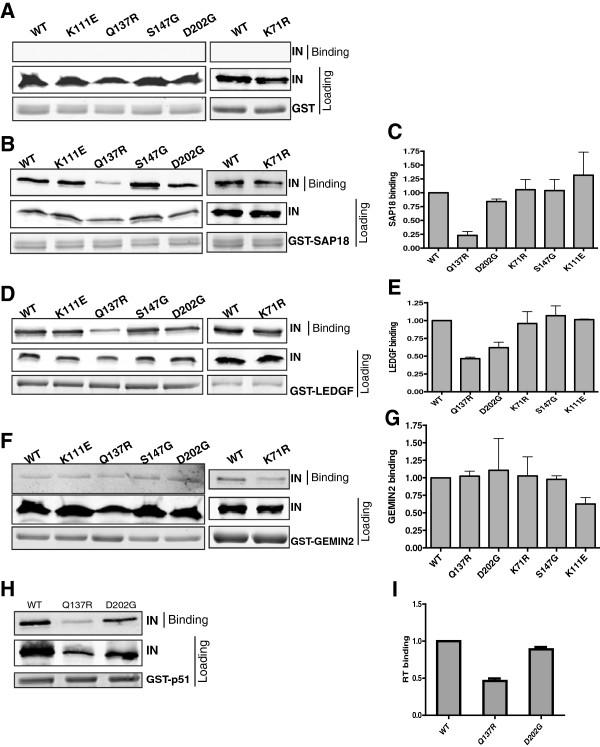
**Protein-protein interactions of IID-IN mutants with RT and various IN binding host factors.***In vitro* pull-downs of IID-IN mutants with GST-tagged IN binding partners were carried out using His_6_-tagged IN (WT or IID-IN mutants) and GST **(A)**, GST- SAP18 **(B** and **C)**, GST-LEDGF **(D** and **E)**, GST-GEMIN2 **(F** and **G)**, and GST-RT **(H** and **I)**. 5μg of GST-tagged proteins were used for each pull-down reaction. Left side panels **(A**, **B**, **D**, **F** and **H)** illustrate Western and Coomassie stained gel to indicate amount of loaded and bound proteins from a representative experiment. Right side panels **(C**, **F**, **G** and **I)** illustrate graphic representation of relative binding of IID-IN mutants compared to that of WT. Quantitation of binding was carried out by normalizing against total IN loaded for each reaction **(C**, **E**, **G**, **I)**. The graphs **(C**, **E**, **G** and **I)** represent averages of three independent experiments (Mean +/- SEM).

Since IID-IN mutants were blocked for reverse transcription and integration, we sought to determine if they were also impaired for binding to other IN-interacting proteins such as LEDGF, GEMIN2, and SAP18 [29,38,39]. His_6_-tagged WT and IID-IN mutants were each tested for their ability to bind to GST-tagged host proteins. Beads bound to GST alone failed to pull down His_6_-tagged IN proteins (Figure 7A). All IID-IN mutants bound to GST-SAP18 at levels comparable to WT, except for Q137R which exhibited ~4-fold decrease in SAP18 binding (Figure 7B and C). The IID-IN mutants bound LEDGF close to or at WT levels (Figure 7D and E). None of the mutants displayed defects in binding to GEMIN2 (Figure 7F and G). Previous results demonstrated that IN binds to RT and that both p66 and p51 are able to bind to IN equally efficiently *in vitro*[35]. Since highly defective mutants, Q137R and D202G were defective for *in vivo* reverse transcription, these mutants were also tested for their ability to bind GST-RT-p51 and GST-RT-p66. Our results indicated that while Q137R displayed ~45% RT binding, D202G bound RT at levels close to WT (~80% binding compared to WT) (Figure 7H and I). In summary, among the IID-IN mutants, Q137R showed defect in binding to SAP18, LEDGF, and RT but no defect in binding to GEMIN2. Interestingly, the mutant D202G, one of the highly defective IID-IN mutants, exhibited significant binding to all tested host factors including SAP18, LEDGF and GEMIN2, as well as to RT, indicating that the strongest defect observed for this mutant correlated with its defect in binding specifically to INI1.

## Discussion

Many IN mutants exhibit defects at stages other than integration including uncoating, reverse transcription, nuclear localization, assembly, and particle production [5-15]. These pleiotropic defects of IN mutants are poorly understood. However, since IN binds to various cellular proteins, we hypothesized that at least some of these pleiotropic effects could be explained by the inability of mutants to interact with host factors required for various stages in the HIV-1 life cycle. The results of our analysis of IN mutants defective for binding to INI1 (IID mutants) indicate that lack of binding to INI1 could lead to defects in many steps of HIV-1 replication. We have summarized the results of our analysis in a table providing the % activity of the mutants relative to wild type IN at various stages of HIV-1 replication (Table 2).

**Table 2:** Summary of effect of IID-IN mutations on various stages of HIV-1 replication relative to that of the wild type

**Mutation**	**% activity of mutants relative to wild type (average of three experiments)**^**a**^						
	**Binding to INI1**	**Infection in CEM-GFP (day 10)**	**Infection in HeLa (24 hr.)**	**Early RT at 6 hr**	**Late RT at 6 hr**	**2LTR circle formation (at 12 hr.)**	**Integration (at 24 hr.)**
IN (WT)	100	100	100	100	100	100	100
H12Y	20.60	0.12	0.07	11.12	7.15	0	0
Q137R	10.00	0.09	0.40	29.89	12.10	30.62	5.42
D202G	32.00	0.09	0.14	14.71	14.74	48.58	1.34
K71R	46.00	3.74	2.52	38.13	27.73	150.78	61.64
S147G	77.00	29.83	6.93	74.01	124.82	164.75	33.29
K111E	ND	29.26	4.68	71.25	104.25	47.07	65.30
D116A	ND	0.11	3.36	79.39	60.15	13369.26	1.44

^a^ % activity of mutants was determined by considering the activity of wild type as 100%.

The parent residues of IID-IN mutations are located largely along one face of the IN monomer and map primarily to the catalytic core domain, corroborating the previous idea that core domain is the major site of INI1-IN interaction [20]. Many of the IN residues identified as being involved in INI1 binding are highly conserved among HIV-1 and SIV_cpz_, but are less conserved among IN proteins from other lentiviruses, including SIV_mac_, HIV-2 and HTLV-1. Specifically, the D202 residue is invariant among HIV-1 and SIV_cpz_, and this residue is replaced by an Asn (N202) at the same position in the majority of other primate lentiviruses. The invariant nature of the D202 residue in HIV-1 and SIV_cpz_, the presence of a different residue, N202, in the other primate lentiviruses, combined with the observation that INI1 binds to HIV-1 IN but not to other lentiviral INs, raises the possibility that D202 is a key residue which determines interaction of INI1 with HIV-1 IN. Future crystallographic and molecular studies might shed light on this hypothesis, which are beyond the scope of this report.

Our observation indicates that the degree of IN-INI1 binding correlates well with the level of viral replication, in that mutants H12Y, Q137R and D202G, severely impaired for INI1 binding, are dramatically impaired for replication in both multi- and single-cycle infection assays (Figure 2). While Q137R exhibits defect in several protein-protein interactions, D202G is not significantly defective for interaction with WT IN or with the other known IN-binding proteins tested (LEDGF, SAP18, GEMIN2 or RT, Figure 7), but is severely defective for binding to INI1 (Figure 1). These mutants were highly defective for early and late reverse transcription *in vivo.* However, the mutant virions exhibited no defects either in reverse transcriptase activity or the uncoating activity *in vitro* (Figure 6). Electron microscopic analysis of mutant particles indicated that H12Y and Q137R mutants have highly aberrant virion morphology. While H12Y mutants exhibit immature virion morphology, Q137R and D202 virions exhibit varying degree of defects in morphology and accumulate mixture of virions that have acentric crescent shaped, or immature or aberrant cores (Figure 5). These results indicate that mutants that are severely defective for binding to INI1 exhibit aberrant virion morphology.

It is interesting to note that while the capsid morphology is affected by the presence of IID-IN mutations, these capsids are stable. The relationship between the morphology and stability of the capsid has not been extensively studied. There are examples in the literature, where CA (p24) mutants show aberrant morphology due to altered stability of the capsid [40,41]. There are several reports of the presence of aberrant morphology of capsids when IN protein is mutated (class II IN mutants). However, these studies do not report the capsid stability. Furthermore, in a report using C130S IN mutation it was demonstrated that capsid was unstable [5], but the morphology of the mutant capsid was not described. Here we have studied both capsid morphology and stability, and have found that mutations in IN can alter the morphology but not the stability of the capsid. We surmise that mutations of residues within CA can alter the intermolecular interactions and thereby affect the stability of the capsid in addition to altering its shape. However, mutations in IN may influence protein-protein interactions during capsid assembly and maturation, causing abnormal morphology but not necessarily affect the intermolecular interactions within the mature capsid. Therefore, our results, albeit unexpected, suggests that it is possible for a mutant virus particle to have a morphologically aberrant yet intrinsically stable capsid.

Our results indicate an interesting trend with respect to late RT step with the mutants H12Y, Q137R and D202G. These mutants demonstrate a higher second strand synthesis activity at 2 hr time point when compared to that of wild type. The activity of these mutants peaks at 2 hr time point, and shows a precipitous decline at 6 hr time point. On the contrary, the activity of wild type DNA synthesis shows a lag phase at 2 hr time point, and shows a peak activity at 6 hr time point. This result suggests that in the mutants, viral DNA synthesis may start prematurely, but is unstable and hence the DNA gets degraded. This phenotype indicates a role for IN-INI1 binding in preventing the premature second strand synthesis and for stabilizing the late RT products. However, further work is needed to understand the mechanism underlying this differential kinetics of RT (Figure 3, Panel D). K71R, on the other hand, appears to have an overall low level of DNA synthesis.

At this point we cannot determine if INI1 has a direct role in reverse transcription. The effect of IID-IN mutations could be indirect and could affect the protein folding leading to defects in assembly, morphogenesis and reverse transcription. However, it is interesting to note that the phenotype we observe for D202 and other IID-IN mutants are similar to the phenotype observed for particles produced in MON cells, the INI1-/- rhabdoid tumor cells [26]. The HIV-1 particles produced in MON are severely impaired for infection with a block occurring at or prior to reverse transcription. These studies indicate that lack of INI1 in the producer cells makes the particles defective for reverse transcription. While these studies argue for a role of INI1 and/or INI1-associated complexes in reverse transcription, further studies are required to determine the effect of INI1 on particle assembly and morphogenesis. Since INI1 is likely to bind to the IN portion of Gag-Pol, these studies may uncover a specific and distinct role of Gag-Pol during late events.

Partially defective IID-IN mutants, S147G, and K111E were less defective for reverse transcription and 2LTR circle formation, suggesting that partial retention of INI1 binding is enough to proceed through these steps. These mutants did not exhibit any defect in binding to other host factors. The partially defective mutants however were defective for integration. The defect in integration is partial and correlated with the degree of defect of these mutants for binding to INI1. Interestingly, both S147G and K111E retain their *in vitro* integration activity, while the highly defective mutants either did not express stable proteins in bacteria or were defective for integration activity (data not shown). However, the observed results obtained with partially defective IN mutants are consistent with the idea that IN-INI1 interaction is necessary for integration *in vivo*. The IN-INI1 interaction has been strongly implicated in recent findings as a necessary component of full-site integration into chromatinized DNA *in vitro*[28]. It was reported that both SWI/SNF chromatin remodeling activity and the presence of INI1 in the active SWI/SNF complex, are required for efficient and competent integration in an *in vitro* nucleosome model [28]. These results suggest that binding of IN to INI1 within the SWI/SNF complex is required for integration into chromatinized target. Our *in vivo* results support this finding and strongly suggest that full binding between IN and INI1 are necessary to efficiently complete integration. Future studies identifying mutants that are specifically defective for INI1 binding and those that are not defective for integration *in vitro*, are likely to shed light on the role of INI1 in integration.

The question is how does INI1 play a role in assembly, reverse transcription and integration of HIV-1. One possibility is that binding of INI1 to Gag-Pol could allow proper assembly and morphogenesis. Subsequent to particle production, the IN-INI1 interaction is maintained in the virions and is then carried to the target cell where it could influence reverse transcription in the cytoplasm and integration in the nucleus. INI1 has been found in the purified and subtilisin treated HIV-1 virions and it has been found in purified reverse transcription complexes within the infected cells [42]. INI1 has multiple cellular functions as indicated by numerous protein-protein interactions, non-specific DNA binding. It is possible that specific functions of INI1 may affect distinct stages of the HIV-1 life cycle. Assembly of Gag-Pol with INI1 and its incorporation into virions along with associated SAP18 and HDAC1 may play a role in early reverse transcription. Indeed, we have demonstrated that incorporation of catalytically inactive mutants of HDAC1 into HIV-1 virions leads to defect in reverse transcription [29]. Subsequently, INI1 may assist IN with integration in the nucleus. Our previous studies have demonstrated that INI1 mutants defective for dimerization and DNA binding activity exhibited reduced activity to stimulate *in vitro* integration, and defect in DNA binding correlated to their impairment in stimulating integrase activity *in vitro*[43]. These studies suggest that DNA binding activity of INI1 may be required for integration. Based on these observations, we speculate that while the ability of INI1 to mediate protein-protein interactions may be important to facilitate late events of HIV-1 replication and proper particle morphogenesis, once INI1 is associated with IN, its ability to bind to DNA may be important to facilitate integration. Thus, distinct functions of INI1 may contribute to different steps of HIV-1 replication. Therefore, lack of INI1 or lack of interaction with INI1 may lead to multiple blocks to HIV-1 replication.

One of the mutants recovered during our reverse two hybrid screen was K71R; this mutant was previously identified as an INI1-interaction defective mutant of IN [31]. This study indicated that K71R mutant was more replication competent than wild type. However, in our hands, multiple assays testing for replication (multiday replication assay, single cycle infection assay, early and late reverse transcription assays and *in vivo* integration assays) of this mutant indicated it to be partially defective. We noted that during multiday replication, many of the mutants readily reverted to wild type after the first round of replication. For example, S147G, a partially defective mutant showed slow replication in the first round of replication in a multiday replication assay. However, continued cultures for more than 14 days resulted in increased replication. Sequence analysis of the virions from this culture indicated that S147G virus has reverted to true wild type (data not shown). Therefore, we speculate that either difference in the assays used in our two studies or reversion could account for the observed differences in the two results.

In summary, we find that IN mutants defective for binding to INI1 exhibit defects at early post-entry events and at integration. The observed defects at reverse transcription are consistent with the previous observations of the effects of lack of INI1 or mutations in INI1-associated HDAC1 [26,29]. Furthermore, the highly defective mutants exhibited aberrant virion morphology. These results suggest that interaction of IN within the context Gag-Pol with INI1 is necessary for proper assembly and virion morphogenesis. Defects in these events could manifest as defects in early reverse transcription. However, the partially defective IID-IN mutants that have normal virion morphology are partially defective for DNA synthesis and integration. The genetic studies carried out thus far indicate that disrupting IN-INI1 interaction results in dramatic inhibition of HIV-1 replication: (1) sequestering IN by using a dominant negative mutant of INI1 results in potent inhibition of late events [21]; (2) INI1-/- cells are defective for particle production and those particles that are produced are defective for early events [26]; (3) IN mutants that are defective for INI1 binding result in dramatic effects in HIV-1 replication (the current study). Since strong disruption of the IN-INI1 interaction leads to multiple blocks at reverse transcription and integration, the virions are severely compromised for replication.

## Conclusions

We have found that IN mutants defective for binding to INI1 exhibit defects in particle morphogenesis, early post entry events and/or integration. We propose that INI1 binding to Gag-Pol facilitates proper assembly of the complex, which is required for subsequent post-entry events in the target cells. Thus, we propose that disrupting IN-INI1 interactions may lead to the inhibition of multiple steps of viral replication.

## Methods

### Cells and reagents

293 T and HeLa cells (ATCC) were cultured in Dulbecco’s modified Eagle medium (DMEM) and supplemented with 10% FBS, L-glutamine, and Pen-strep. CEM-GFP cells were grown in RPMI1640 containing 10% FBS, L-glutamine, Pen-strep and supplemented with 500 μg/mL G418.

### Isolation of IID-IN mutants by using reverse yeast two-hybrid system

A random mutation library of HIV-1 IN was generated by using PCR-based mutagenesis utilizing the error-prone nature of TaqMan polymerase [30]. Specifically, a two-hybrid plasmid vector carrying wild type IN fused to the GAL4-Activation domain (GAL4-AD in plasmid pGADNot-IN [30,44], was mutagenized by using TaqMan polymerase-based PCR mutagenesis system. The mutagenized plasmid pool was co-transformed into yeast with a plasmid expressing INI1 fused to LexA DNA Binding domain (LexADB-INI1 in plasmid pSH2-INI1 [23]. A panel of INI1-interaction defective IN (IID-IN*) mutants were isolated that resulted in white colonies in the X-gal assay. Plasmid harboring mutant IN (pGADNot-IID-IN*) isolated from yeast, were retransformed into yeast along with pSH2-INI1 to confirm the interaction-defective phenotype. The clones were sequenced to determine the presence of mutations.

### Generation of HIV-1_NL4-3_ full-length and HIV-Luc viral clones harboring IID-IN mutations

HIV-1_NL4-3_ viral clones containing IID-IN mutations were generated in two stages. First, a 2.3 Kb Age I (position 3485 bp) and Sal I (position 5785 bp) fragment of HIV-1_NL4-3_ containing the IN open reading frame was subcloned into the pEGFPN1 vector to generate an intermediate vector pEGFPN1-IN-int. IID-IN mutations were introduced into pEGFPN1-IN-int using the QuickChange II Mutagenesis Kit (Stratagene). The Sal I/Age I fragment containing the desired mutation was subsequently cloned into pNL4-3 as well as pNL4-3.Luc.R-E-(HIV-Luc) to obtain mutant viral clones for use in multiday and single cycle infection assays.

### Construction of YFP-IID-IN mutant expression plasmids

IID-IN mutants were introduced into a previously described vector encoding IN fused to YFP [25] using QuickChange Lightening site-directed mutagenesis kit (Stratagene) and the following primers for each mutant: H12Y (Forward 5’-GCCCAAGATGAATATGAGAAATATCACAGTAATTGGAGAGCAATGGC-3’) (Reverse 5’-GCCATTGCTCTCCAATTACTGTGATATTTCTCATATTCATCTTGGGC-3’); Q137R (Forward 5’-GGGCGGGAATCAAGCGGGAATTTGGAATTCCC-3’) (Reverse 5’-GGGAATTCCAAATTCCCGCTTGATTCCCGCCC-3’); K71R (Forward 5’-CATTTAGAAGGACGAGTTATCCTGGTAGCAGTTCATGTAGCC-3’) (Reverse 5’-GGCTACATGAACTGCTACCAGGATAACTCGTCCTTCTAAATG-3’); S147G (Forward 5’-CCCTACAATCCCCAAGGTCAAGGAGTAGTAGAATCTATG-3’) (Reverse 5’-CATAGATTCTACTACTCCTTGACCGTTGGGATTGTAGGG-3’); K111E (Forward 5’-GGAAGATGGCCAGTAGAAACAATACATACAGACAATGGC-3’) (Reverse 5’-GCCATTGTCTGTATGTATTGTTTCTACTGGCCATCTTCC-3’). YFP-D202G (Forward 5’-GCAGGGGAAAGAATAGTAGGCATAATAGCAACAGAC-3’) (Reverse 5’-GTCTGTTGCTATTATGCCTACTATTCTTTCCCCTGC-3’).

### Construction of plasmids expressing 6His-IID-IN

To construct pQE32-IID-IN* mutant plasmids expressing 6His-tagged IID-IN mutants, the GFP-IN constructs were used as template and IN sequences were PCR amplified using the following primers containing *Bam* HI and *Sal* I restriction sites: YFP-C3-INForward 5’-GGTACCGCGGGCCCGCGGATCCACTTTTTAGATGGAATAGATAAGGCCC-3’ and YFP-C3Reverse 5’-GATCCGGTGGAAGATCTGTCGACCTTAATCCTCATCCTGTCTACTTGC-3’. The PCR product was cloned into the pQE32 vector (Qiagen) and transformed into *E. coli* strain M15 (pDM1) for expression of protein.

### Purification of 6His-IID-IN mutants and GST-tagged proteins

1 L cultures of 6His-IID-IN mutants were grown to an OD600 of ~0.8 before induction with 1 mM IPTG for 3-6 hours at 37°C. The cell pellet from 1 L of culture was resuspended in 20 mL of lysis buffer A (50 mM NaPO4 pH 7.4, 0.1 mM EDTA, 5% glycerol, 10 mM β-ME, 1 mM PMSF) and spun at 17,000 × g for 10 min. Lysozyme was added to a final concentration of 1 mg/mL and incubated for 30 min at 4°C before adding NaCl to a final concentration of 1 M. Lysates were incubated 30 min at 4°C, then sonicated three times for 1 min each, with cooling intervals. Sonicated lysates were then passed through a 21G1.5 syringe needle twice and centrifuged at 52,000 × g for 30 min at 4°C. Clarified supernatant was then collected and used for binding assays.

GST, GST-INI1, GST-IN, GST-RT-p51 and GST-SAP18 proteins bound to G-beads were prepared as previously described [23]. The GST-p51 construct was prepared by PCR amplifying RT sequence from HIV-1_NL4-3_ using the following primers containing *Bam* HI and *Eco* RI restriction sites: (p51 Forward 5’-CCGGGATCCCGCCCATTAGTCCTATTGAGACTGTACCAGTA-3’), (p51 Reverse 5’-CGGAATTCGAAAGTTTCTGCTCCTATTATGGG-3’). GST-LEDGF (a gift from Dr. Alan Engelman) and GST-GEMIN2 (a gift from Dr. Gideon Dreyfuss) were purified in a similar manner as described above with slight differences. GST-LEDGF in BL21 cells were grown to an OD600 ~0.9 at 37°C before induction with 0.5 mM IPTG for an additional 3 hours at 28°C. Pellets from 250 mL cultures were resuspended in 10 mL of low salt buffer: 50 mM NaCl, 50 mM Tris-Cl pH 7.5, 1 mM EDTA, 0.5% IGEPAL, 5 mM DTT, and one Complete mini protease inhibitor cocktail tablet (Roche) per 10 mL buffer. GST-GEMIN2 was isolated similar to GST-LEDGF with slight differences in low salt lysis buffer (50 mM NaCl, 25 mM Tris-Cl pH 7.5, 1 mM EDTA, 2 mM DTT, protease inhibitor tablet).

### GST pull-down assays

5 μg of GST-tagged proteins bound to glutathione sepharose 4B beads were incubated for 1 hour at 4°C with normalized amounts of either clarified bacterial cells lysates containing 6His-WT-IN and 6His-IID-IN mutants in binding buffer (20 mM HEPES-KOH pH 8.0, 5 mM DTT, 0.1% IGEPAL, protease inhibitor tablet) or mammalian cell extract expressing YFP-fusions of IID-IN mutants. Following incubation, beads were washed 5-7 times with buffer containing 50 mM Tris-Cl pH 8.0, 0.1 mM EDTA, 200 mM NaCl, 0.5% IGEPAL, 25 mM PMSF. Bound proteins were separated by SDS-PAGE and analyzed using indicated antibodies. The relative binding was quantified by using densitometry (Odyssey infrared imaging system, LI-COR). Briefly, the fraction of protein bound was determined for each clone compared to the signal in the loading control. Relative binding was determined by comparing the binding ability of mutants to that of the wild type protein.

To test binding of IID-IN mutants to INI1, 293 T cells were transfected with 5 μg YFP-IN or YFP-IID-IN mutant DNA (in pEYFP-C3 vector). Whole cell lysates were collected 48 hours post-transfection and normalized for YFP-IN levels using Western blot probed for IN. Clarified lysates, containing normalized levels of YFP-IN or YFP-IID-IN, were then incubated with 5 μg of GST-tagged INI1, bound to glutathione sepharose 4B beads, and incubated for 1 hour at 4°C. Pull-down reactions were performed as described above.

### Virus production

Viral stocks of full-length HIV-1 and HIV-Luciferase (HIV-Luc) were prepared by transient transfection of 50-60% confluent 293 T cell monolayers in 100 mm plates with 10 μg pNL4-3, or cotransfection of 5 μg pNL4-3. Luc. R-E- (deleted for *env*) and 5 μg VSV-G plasmid DNA (courtesy of Dr. Nathaniel Landau, NIH AIDS Research and Reference Reagent Program). Virus was collected 48 hours post-transfection and clarified by passing through a 0.45 μm cellulose acetate filter (Corning). Clarified supernatant was then treated for 30 minutes with 20 units DNaseI (Roche)/mL supernatant at 37°C. Viral stocks were measured for p24 using a p24 enzyme-linked immunosorbent assay (ELISA) (Advanced Bioscience Laboratories).

### Multiday replication

25 ng p24 of each HIV-1_NL4-3_ IID-IN mutant and WT were used to infect 200,000 CEM-GFP cells for 2 hours in a 2 mL culture. After incubation with virus, 18 mL of complete RPMI1640 was added to the culture in a 75 cm^2^ flask. 1 mL of culture was collected every two days for 16 days. Viral replication was monitored by observing cellular GFP levels and by measuring p24 levels in the culture supernatant.

### Luciferase assays

Cells infected with HIV-Luciferase were assayed 24 hours post-infection using the Luciferase Assay System (Promega) following the manufacturer’s instructions.

### Exogenous RT assay

500 ng p24 of HIV-1_NL4-3_ WT and IID-IN mutants were PEG precipitated overnight at 4°C and centrifuged at 16,000 × g, 45 min, at 4°C. Viral pellets were lysed in 12.5 μL solution B (0.9% Triton X-100, 440 mM KCl) and vortexed for 1 minute before adding 50 μL solution A (25 mM Tris pH 7.8, 0.25 mM EDTA, 0.025% Triton X-100, 50% glycerol, 2 mM DTT, 100 mM KCl). 10 μL of each lysate was incubated with 40 μL of an RT reaction cocktail (50 mM Tris pH 8.0, 10 mM DTT, 60 mM NaCl, 10 μg/mL oligo-dT, 100 μg/mL poly-rA, 100 μM dTTP, 1 mCi/μL α^32^P-dTTP, and 10 mM MgCl_2_) for 1 hour at 37°C. 10 μL of each reaction was then spotted on DEAE paper, washed and measured in a scintillation counter.

### Isolation of HIV-1 cores and *in vitro* uncoating assay

Cores were isolated from concentrated virions as previously described [37]. Briefly, virus supernatants from transfected 293 T cells were concentrated by centrifugation at 32,000 rpm (175,000 × g at r_max_) for 3 hours at 4°C. Viral pellets were resuspended in 300 μl of 1X STE buffer (10 mM Tris–HCl [pH 7.4], 0.1 M NaCl, 1 mM EDTA) and incubated at 4°C for at least 2 hours to allow small clumps of virus to disperse. Concentrated virions were then ultracentrifuged (187,000 × g at r_max_ for 16 hours at 4°C) through a 30-70% sucrose gradient overlaid with 1% Triton X-100. Fractions (1 ml) were collected from top of the sucrose gradient and CA content of the fractions was determined by p24 ELISA.

For the *in vitro* uncoating assay, samples of purified wild-type and mutant HIV-1 cores (50 μl, containing ~50 ng of p24) were diluted in 1X STE buffer, pH 7.4 (200 μl). The reactions were then incubated at 37°C for indicated times followed by ultracentrifugation at 45,000 rpm (Beckman TLA-55 rotor, 125,000 × g at r_max_) for 20 min at 4°C. Supernatants were removed and pellets were dissolved in sample diluent (10% Donor Calf Serum and 0.5% Triton X-100 in phosphate–buffered saline). CA content of supernatants and pellets was measured by p24 ELISA. The extent of uncoating was determined as the percentage of CA content of supernatant to the total quantity of CA in the reaction.

### Real-time PCR to detect early and late RT products, 2LTR circles and integrated product

50% confluent 293 T cells were spinoculated with 20 ng p24 of HIV-Luc virus for 2 hours at 15°C and spun at 400 × g. Cells were collected at various time points post-infection and harvested for genomic DNA using the DNeasy Blood and Tissue Kit (QIAGEN). All genomic DNA samples were normalized and 300-500 ng DNA was used per 50 uL real-time PCR reaction containing 300 nM primers, 100 nM probe and 2× Taqman Universal Master Mix (Applied Biosystems). Primers and probes to detect early and late RT products are as follows [45]: Early RT primers, ert2f 5’-GTGCCCGTCTGTTGTGTGAC-3’, ert2r 5’-GGCGCCACTGCTAGAGATTT-3’, and ert2 probe 5’-CTAGAGATCCCTCAGACCCTTTTAGTCAGTGTGG-3’. Late RT primers, MH531 forward 5’-TGTGTGCCCGTCTGTTGTGT-3’ , MH532 reverse 5’-GAGTCCTGCGTCGAGAGAGC-3’ , and LRT probe 5’-CAGTGGCGCCCGAACAGGGA-3’. Cycling conditions on an ABI 7900HT are 2 min at 50°C, 10 min at 95°C, followed by 40 cycles of 15 sec at 95°C and 1 min at 60°C. Primers and probes to detect 2LTR circles are as follows: MH535 forward 5’-AACTAGGGAACCCACTGCTTAAG-3’ , MH536 reverse 5’-TCCACAGATCAAGGATATCTTGTC-3’ , and MH603 probe 5’-ACACTACTTGAAGCACTCAAGGCAAGCTTT-3’.

To detect the integrated provirus, nested Alu-PCR was performed [46]. The products were measured by isolating genomic DNA 24 hours post-infection and performing nested PCR. In the first semi-quantitative PCR round, Alu-gag was amplified as described [46]. Real-time PCR, using the MH531, MH532, and late RT primers and probe, was then performed on products from the first round PCR using conditions described above. Standard curves were generated by isolating genomic DNA from 293 T cells stably infected with HIV-1 containing GFP and a hygromycin-resistance marker [46] and performing nested PCR from this cell line. Ct values were then plotted against dilutions of the hygromycin-resistant HIV-1 plasmid to create a standard curve.

### Western blots

Whole cell extract from 293 T cells transfected with pNL4-3 WT and IID-IN mutant plasmids were collected after lysing with RIPA buffer. Culture supernatant containing virus was centrifuged over a 20% sucrose cushion at 112,000 × g for 2 hours at 15°C. The viral pellet was washed once with PBS before resuspension in PBS and HEPES. Normalized amounts of p24 from each virus prep was precipitated with a 30% PEG8000/0.4 M NaCl solution and resuspended in lysis buffer containing 50 mM Tris-Cl pH 7.5, 150 mM NaCl, 1 mM EDTA, 1% IGEPAL, 1% Triton X-100, 0.5% sodium deoxycholate, 0.5 M PMSF. Viral proteins and cell lysates were resolved by SDS-PAGE and analyzed by Western blot with indicated antibodies.

### Transmission electron microscopy

293 T cells in a 6-well format were transfected with 2ug each of the indicated pNL4-3 either WT or IID-IN mutant plasmids using the Mammalian Cell Transfection kit (Millipore Catalogue #s-001). Media was changed 16 hours post-transfection and cells were fixed 6 hours post-media change with buffer containing 2.5% glutaraldehyde in 0.1 M sodium cacodylate buffer, postfixed with 1% osmium tetroxide followed by 2% uranyl acetate, dehydrated through a graded series of ethanol and embedded in LX112 resin (LADD Research Industries, Burlington VT). Ultrathin sections were cut on a Reichert Ultracut UCT, stained with uranyl acetate followed by lead citrate and viewed on a JEOL 1200EX transmission electron microscope at 80kv.

## Competing interests

The authors declare that they have no competing interests.

## Authors’ contributions

SM carried out majority of the experiments and wrote the manuscript. MN carried out GST pull down assays. XW contributed to virological assays and GST-pull down assays, AP isolated the IID-IN mutants by using reverse yeast two hybrid system. VS carried out capsid stability and uncoating assays. VRP analyzed the results and participated in writing the manuscript. CA supervised capsid stability and uncoating assays and contributed to writing the manuscript. GVK conceived of the study, supervised and participated in the design and co-ordination the study, wrote the manuscript. All authors read and approved the final manuscript.

## Supplementary Material

Additional file 1: Table S1 Surface accessibility of IN residues implicated in binding to INI1/hSNF5. Click here for file

Additional file 2: Figure S1. Effect of IID-IN mutations on HIV-1 viral protein synthesis and particle production. **A.** Levels of virion associated p24 in culture supernatants of cells transfected with HIV-1 WT or IID-IN mutants. **B.** Levels of intracellular p24 in the producer cells transfected with HIV-1 WT or IID-IN mutants. **C.** A graphic representation of p24 release efficiency as determined by the fraction of virion- and cell- associated p24 compared to that of the total p24 based on values in A and B. The values are expressed as percentages of the total. Click here for file
