# Supplementary material for: INI1/hSNF5-interaction defective HIV-1 IN mutants exhibit impaired particle morphology, reverse transcription and integration in vivo
Source: Retrovirology. 2013 Jun 24;10:66. doi: 10.1186/1742-4690-10-66 (PMC3708822; doi:10.1186/1742-4690-10-66)
Supplement: Additional file 1: Table S1 — Surface accessibility of IN residues implicated in binding to INI1/hSNF5. [file 1742-4690-10-66-S1.docx]

| **Supplementary Table 1. Surface accessibility of IN residues implicated in binding to INI1** | | |
| --- | --- | --- |
| **Clone #** | **Residue/s mutated** | **%ASA by PISA*** |
| T1 | W61R | 1.43 |
| T3 | W61R | 1.43 |
| T5 | **K111E** | 99.85 |
| T6 | W61R | 1.43 |
| T7 | I5M  V176A  V201G | 33.86  0.17  83.05 |
| T8 | D253G | 50.95 |
| T18 | **Q137R**  T174A | 63.92  62.08 |
| T19 | S81G | 4.18 |
| T20 | Y22C | 11.92 |
| T117 | **K71R**  G123S  V176A | 89.17  33.38  0.17 |
| T178 | **S147G**  **D202G** | Missing loop  83.58 |

*The % accessible surface area was determined using

IN dimer structure using the program PISA.
