# Supplementary figures and images for: INI1/hSNF5-interaction defective HIV-1 IN mutants exhibit impaired particle morphology, reverse transcription and integration in vivo
Source: Retrovirology. 2013 Jun 24;10:66. doi: 10.1186/1742-4690-10-66 (PMC3708822; doi:10.1186/1742-4690-10-66)

Supplementary Figure 1

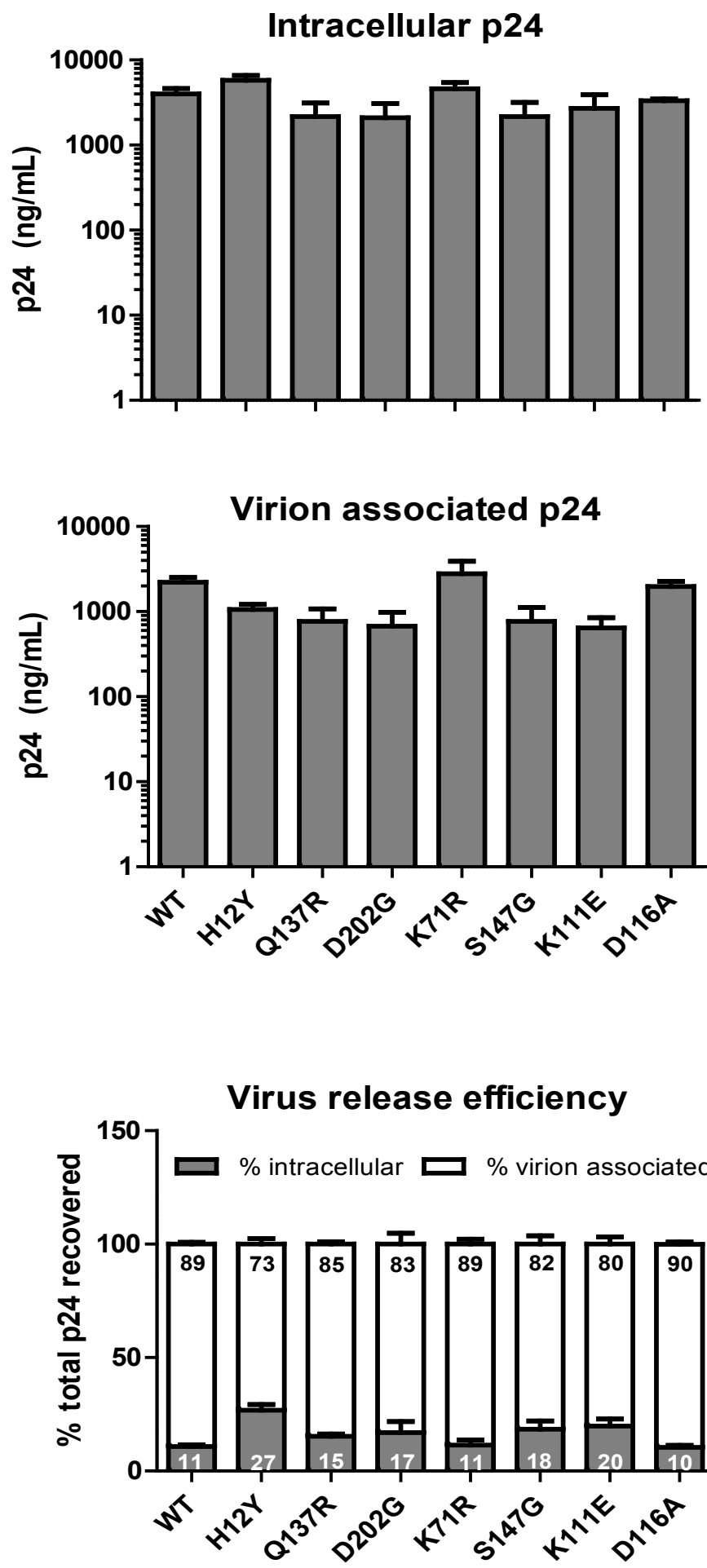

Supplement: Additional file 2: Figure S1. — Effect of IID-IN mutations on HIV-1 viral protein synthesis and particle production. A. Levels of virion associated p24 in culture supernatants of cells transfected with HIV-1 WT or IID-IN mutants. B. Levels of intracellular p24 in the producer cells transfected with HIV-1 WT or IID-IN mutants. C. A graphic representation of p24 release efficiency as determined by the fraction of virion- and cell- associated p24 compared to that of the total p24 based on values in A and B. The values are expressed as percentages of the total. [file 1742-4690-10-66-S2.pdf]
